# Protein Tyrosine Phosphatase Receptor S Acts as a Metastatic Suppressor in Malignant Peripheral Nerve Sheath Tumor via Profilin 1-Induced Epithelial-Mesenchymal Transition

**DOI:** 10.3389/fcell.2020.582220

**Published:** 2020-10-09

**Authors:** Jie-Yi Ren, Yi-Hui Gu, Xi-Wei Cui, Man-Mei Long, Wei Wang, Cheng-Jiang Wei, Bin Gu, Hai-Bing Zhang, Qing-Feng Li, Zhi-Chao Wang

**Affiliations:** ^1^Department of Plastic and Reconstructive Surgery, Shanghai Ninth People’s Hospital, Shanghai Jiao Tong University School of Medicine, Shanghai, China; ^2^Department of Pathology, Shanghai Ninth People’s Hospital, Shanghai Jiao Tong University School of Medicine, Shanghai, China; ^3^CAS Key Laboratory of Nutrition, Metabolism and Food Safety, Shanghai Institute of Nutrition and Health, Chinese Academy of Sciences, Shanghai, China

**Keywords:** PTPRS, MPNST, epithelial-mesenchymal transition, actin-binding proteins, profilin 1

## Abstract

Malignant peripheral nerve sheath tumors (MPNST) are aggressive sarcomas with over half of cases developed in the context of neurofibromatosis type 1. Surgical resection is the only effective therapy for MPNST. The prognosis is very dismal once recurrence or metastasis occurs. Epithelial-mesenchymal transition (EMT) is a key process of recurrence and metastasis involving reorganizations of the actin cytoskeleton and actin-binding proteins (ABP) play a non-negligible role. Protein tyrosine phosphatase receptor S (PTPRS), a tumor suppressor previously reported in colorectal cancer, hepatocellular carcinoma and head and neck cancer, is thought to mediate cell migration and invasion by downregulation of EMT. However, its role in MPNST remains unknown. In the present study, by using tissue microarray we demonstrated low expression of PTPRS was related to poor prognosis in MPNST. Knockdown of PTPRS in MPNST cell lines increased migration/invasion and EMT processes were induced with increased N-cadherin and decreased E-cadherin, which indicated PTPRS may serve as a tumor suppressor in MPNST. In addition, we tested all EMT related ABP and found profilin 1 was significantly elevated in PTPRS downregulated MPNST cell lines. As a member of actin-binding proteins, profilins are regulators of actin polymerization and contribute to cell motility and invasion, which have been reported to be responsible for EMT. Moreover, results showed that downregulation of profilin 1 could restore the EMT processes caused by PTPRS downregulation *in vitro* and *in vivo*. Furthermore, high expression of profilin 1 was significantly associated with dismal prognosis. These results highlighted PTPRS served as a potential tumor suppressor in the recurrence and metastasis of MPNST via profilin 1 induced EMT processes and it might provide potential targets for future clinical therapeutics.

## Introduction

Malignant peripheral nerve sheath tumors are aggressive and devastating tumors of peripheral nerve with an incidence of 0.001 percent in the general population (Mowery and Clayburgh, 2019). However, the risk of MPNST development is significantly elevated in NF1 patients, particularly in those with plexiform neurofibromas (Wei et al., 2020). NF1 associated MPNST consists of over half of total MPNST cases and is the leading cause of death in NF1 patients (Higham et al., 2018). Surgical resection with a sufficient wide margin is currently the only potential curative management for MPNST patients (Reilly et al., 2017). However, many patients lose the chance of surgical resection at the time of diagnosis due to MPNST strong metastasis potential. Moreover, the local recurrence rate remains high after surgical resection and traditional chemo/radio therapies show mild benefits (Hirbe et al., 2017; Pasquali and Gronchi, 2017). Therefore, investigating underlying mechanisms of MPNST recurrence and metastasis is of great importance to develop future target therapies and improve prognosis.

Protein tyrosine phosphatase receptor S is a member of distinct family of leukocyte common antigen-related receptor-type phosphatases (Bunin et al., 2015). The physiological role of PTPRS has been well-established in the development of nerve system (Meathrel et al., 2002; Stewart et al., 2013) and pituitary gland (Wallace et al., 1999). Recently, studies have revealed vital roles of PTPRS in immune-mediated intestinal inflammation (Murchie et al., 2014), autophagy and progression of various types of cancers (Wang et al., 2015). The role and underlying mechanisms of PTPRS in different diseases show significant heterogeneity (Du and Grandis, 2015). In hepatocellular carcinoma and head and neck cancer, PTPRS regulated EGFR in EMT processes and drug resistance (Lui et al., 2014). Whereas, in colorectal cancer PTPRS regulated RAS pathway activity by inactivating ERK and preventing its nuclear translocation (Davis et al., 2018). However, the role of PTPRS in neurogenic tumors remains unclear, especially there is previously no study investigating its role in MPNST. Our present study is the first one demonstrating the vital role of PTPRS in MPNST recurrence and metastasis.

Epithelial-mesenchymal transition is a fundamental biological process during which epithelial cells acquire mesenchymal characteristics (Mittal, 2018). EMT plays a vital role in cancer recurrence and metastasis (Mittal, 2018). The dramatic changes in phenotypes and biological behaviors involve a reorganization of actin cytoskeleton, which leads to gain of cellular plasticity and enables membrane protrusions for invasive growth (Yin et al., 2020). It is well established that the organization and dynamics of actin cytoskeleton are controlled by a large array of ABP (Lappalainen, 2016). However, the key ABP in PTPRS mediated MPNST recurrence and metastasis remains unknown.

To explore the clinical impacts of PTPRS and its downstream vital ABP in EMT processes in MPNST recurrence and metastasis, correlation studies of clinicopathological characteristics using tissue microarrays and biological functional assays *in vitro* and *in vivo* have been conducted. In addition, all EMT related ABP have been screened and profilin 1 is the potential key ABP in PTPRS mediated EMT processes, which expression significantly correlates with dismal prognosis of MPNST patients. Taken together, our study shows PTPRS acts as a metastatic suppressor in MPNST via profilin 1-induced EMT and PTPRS and profilin 1 might be potential promising biomarkers for target therapy.

## Materials and Methods

### Cell Lines

One HSC line and two MPNST cell lines (sNF02.2, sNF96.2) were purchased from ATCC (American Type Culture Collection, ATCC). Five MPNST cell lines (ST88-14, STS26T, T265, S462, and S462TY) were kindly granted by Prof. Vincent Keng (Li X.X. et al., 2018) and Prof. Jilong Yang (Du et al., 2013). All MPNST cell lines were derived from NF1 patients, except STS26T. Cell lines were maintained in DMEM, 10% FBS and penicillin/streptomycin (Gibco, United States) and were tested mycoplasma negative every 3 months. Verification of cell lines was confirmed by Short Tandem Repeat DNA profiling (Applied Biological Materials Inc., Canada).

### Patients and Specimens

Fresh human MPNST tissues were gained from surgical resection specimens of patients in Shanghai Ninth People’s Hospital (Shanghai, China). Human NNTs were obtained from surgical resection residuals of patients in facial nerve repair. The study was approved by Ethics Committee of Shanghai Ninth People’s Hospital, Shanghai Jiao Tong University School of Medicine, and informed consent was achieved from patients under institutional reviewer board protocols.

Paraffin-embedded tumor tissues from 49 patients after surgical treatment of MPNST between 2005 and 2018 were used for the construction of tissue microarray and IHC. Tissue microarray scores were independently assessed by two independent researchers. In order to eliminate the error caused by different observation conditions, the researcher was asked to finish the assessment of a total microarray within a continuous time interval about 2 h. The score was determined by the proportion of positive cells so that negative, <25%, 25%∼50%, >50% were, respectively, recorded as 0, 1, 2, 3. Discussion with a third researcher if disagreements occur. Clinical information was collected through review of electronic medical records. 29 of 49 patients’ survival status were accessed and confirmed by telephone follow-up, which data were used for survival analyses. 20 in 49 patients lost follow-up currently and were not be able to be contacted with multiple methods.

### *In vitro* Cell Function Experiments

Cell proliferation experiments were conducted by using Cell Counting Kit-8 (CCK8, Dojindo, Japan). A quantity of 10^3^ cells per well were seeded appropriately in 96-well plates, and CCK8 solution diluted 1:10 by serum-free DMEM was added on day 1, 2, 3, 4, 5, 6, and 7 to measure the 450 nm OD value after 2 h incubation. Every assay was repeated independently for three times.

A scratch assay was used to imitate the process of migration of adherent tumor cells. Cells were seeded and grown to nearly confluence in 6-well plates. Before scratching, cells were starved for at least 12 h by maintaining in 1% serum DMEM. Each scratch was made along the centerline of wells by using 200 uL pipette tips. After softly wash away the floated cells, images of scratch at different intervals were taken by an inverted microscope. The area inside the scratch was measured using ImageJ. Percentage of wound closure = (area of scratch on 0 h − area of scratch on 18 h or 24 h)/area of scratch on 0 h.

Transwell assays were performed using hanging cell culture inserts (8 μm pore size; Millipore, United States). A total of 6 × 10^4^ cells per well were seeded on top compartment in 200 uL serum-free DMEM and gently placed in 24-well plates which were added 600 uL 15% serum DMEM in advance. In case of invasion assays, the top compartments were coated by Matrigel (BD Biosciences, United States) diluted 1:8 in DMEM. The migrated and invasive cells were stained with 0.5% crystal violet and the upper cells were softly removed with a cotton swab after 8 h incubation for migration and 24 h for invasion. Images of four random views were captured, and the cell numbers were counted using ImageJ.

### Quantitative PCR

Total tissue and cell RNA were extracted according to the procedure of the RNeasy kit (Qiagen, Canada). The cDNA was transformed using PrimeScript RT Master Mix Kit (Takara, Japan). Quantitative PCR was performed on cDNA using SYBR Green System (Applied Biosystems). GAPDH was used as an endogenous control. Relative expression was calculated using the ΔΔCT, *n* = 4.

### Western Blottings

Tissues and cells were lysed in a RIPA buffer supplemented with protease and phosphatase inhibitors (Beyotime, China). Protein concentrations were determined with the BCA Protein Assay reagent (Beyotime, China). Antibodies used were Profilin 1 (Abcam, ab124904), PTPRS (Abnova, H00005802-M01), E-cadherin (Abcam, ab1416), N-cadherin (Abcam, ab76011), Snail (Abcam, ab53519), Slug (Abcam, ab51772), a-SMA (Abcam, ab7817), Vimentin (Abcam, ab92547), GAPDH (CST, 2118), β-actin (CST, 3700). Band signals were detected using an Amersham Imager 600 (General Electric Company, Boston, MA, United States).

### Knockdown and Overexpression Experiments

For lentiviral shRNA infection, MPNST cells at 70% confluence were infected with lentiviral particles containing shRNAs targeting PTPRS (target: shA, CCTATTACGTCATCGAATATA; shB, CCAGAGCTATTTCATTGTGAT; shC, GGCTGAAGCTGGATAAGAA) and profilin 1 (target: shA, GCTCCAAGATCTCTAATGTAC; shB, GACCAGTATTGTGTTCCTTGT; shC, GAAGTTGCTGAACTGCATTAC) at a MOI of 10. A random nonsense targeted sequence was used as NC. These were purchased from Hanyin Biotechnology (Shanghai, China). Cells were infected overnight and stable cell populations were selected with puromycin (2 μg/mL) 48 h later. For overexpression plasmid transfection, cells were precultured by antibiotic-free DMEM overnight and replaced with fresh complete medium at 6 h after PTPRS plasmids (purchased from ORIGENE, cat # RC221440) infection. An empty pcDNA was used as NC. Transfected cells were validated using qPCR and western blottings.

### Histological Staining

Tissue sections from patients and mice were stained with hematoxylin and eosin. For IHC, sections were dewaxed in xylene, rehydrated through decreasing concentrations of ethanol. After quick wash, antigens were unmasked and retrieval by microwave in citrate buffer. The primary antibody was applied for overnight incubation at 4°C. After incubation with appropriate biotin-conjugated secondary antibody for 2 h at routine time, the signal was detected with DAB substrate (Vector Laboratories). Sections were counterstained with hematoxylin. Antibodies used were profilin 1 (Abcam, ab124904), PTPRS (Abcam, ab222798).

### Xenograft Tumor and Lung Metastatic Mice Models

16 and 32 male NOD-SCID IL-2 receptor gamma null (Payne et al., 2019) mice (purchased from Shanghai Model Organisms, China) were used, respectively, for xenograft tumor models and lung metastatic models. For the xenograft models, a total of 5 × 10^6^ cells in 100 uL PBS containing 50% Matrigel (BD Biosciences, United States) were injected subcutaneously into the armpit of each mouse. For the lung metastatic mice models, cells were resuspended in 200 uL PBS and then injected via tail vain with 4 × 10^6^ cells per injection. Tumors in this study were allowed to grow in a donor mouse to a maximum volume of approximately 1,000 mm^3^. Size of tumors and weights of mice were measured twice a week. Tumor volume was calculated as follows: *L* × *W*^2^/2, where *L* is the length and *W* is the width. The number of metastatic nodules were calculated by hematoxylin and eosin stained after preparing sections of lung tissues every 50 um. The xenograft models were sacrificed after 7 weeks and the lung metastatic models were sacrificed after 2 weeks. All procedures were performed in accord with the guidelines established by the Shanghai Medical Experimental Animal Care Commission.

### Statistical Analysis

The count of cells and quantitation of western bands were analyzed using ImageJ software. Analyses were performed using SPSS Statistics 23 and GraphPad Prism version 8.0. Univariate Kaplan Meier analyses used a Gehan–Breslow–Wilcox log-rank test. Clinicopathological correlation were analyzed by Spearman correlation. *In vivo* and *vitro* data were shown as mean ± SD and were analyzed by paired or unpaired *t*-test, two-sided. Linear regression was conducted in GraphPad Prism. *p*-value <0.05 was considered significant.

## Results

### PTPRS Is Significantly Downregulated in MPNST

**FIGURE 1:**
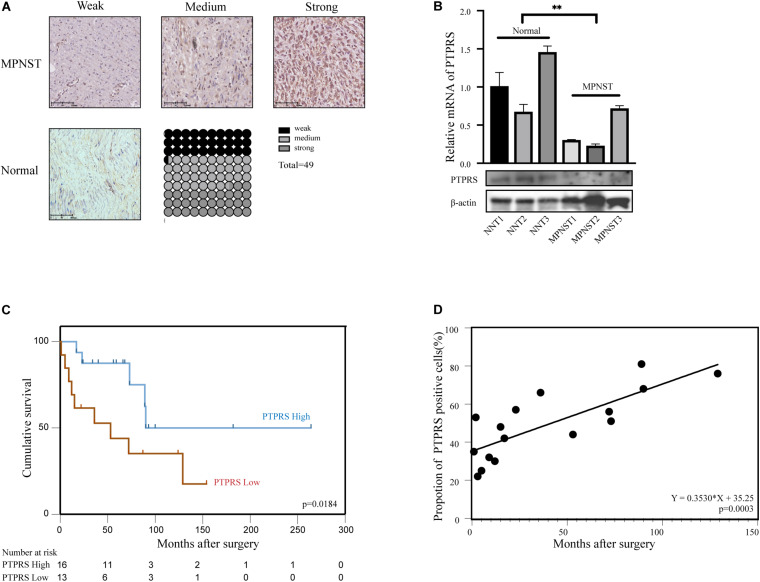
Downregulation of PTPRS correlated with poor prognosis in MPNST patients. **(A)** Representative immunostaining images of PTPRS weak/medium/strong and normal nerve tissue cases and proportions (%) of different levels of tissue microarray dots. **(B)** Relative PTPRS mRNA and protein levels in randomly selected cases of normal nerve tissues and MPNST tissues. β-actin was used as a loading control. **p < 0.05. **(C)** Kaplan–Meier’s curves for cumulative survival according to PTPRS level in MPNST cohort with available follow-up data (*n* = 29, *p* = 0.0184). PTPRS high referred as medium and strong stainings with a score 2 or 3. PTPRS low referred as negative and weak stainings with a score 0 or 1. **(D)** Correlation between expression of PTPRS positive cells in tissues of dead patients and survival time after surgery (*p* = 0.0003). PTPRS, protein tyrosine phosphatase receptor S; MPNST, malignant peripheral nerve sheath tumor; NNT, normal nerve tissue.

To explore the expression of PTPRS in MPNST, IHC stainings of PTPRS in a MPNST cohort including 49 patients were performed. Representative PTPRS IHC stainings of MPNST and normal nerve were shown in Figure 1A. High expression of PTPRS in normal nerve was detected, however, 67.5% MPNST patients showed low or medium PTPRS expression. Consistent with IHC results, western blottings showed significantly high expression of PTPRS in normal nerve compared to MPNST tissue specimens (Figure 1B).

### Low Expression of PTPRS Correlates With Disease Progression and Dismal Prognosis

By classifying MPNST patients into PTPRS high (2–3 scores) and low (0–1 scores) expression group, clinicopathological correlation analyses were performed. The ratio of PTPRS low expression was significantly high in MPNST patients younger than 45 years old (Table 1). Mowery and Clayburgh (2019) demonstrated previously young age below 40 years old significantly correlated with short survival compared to patients with age over 40 years. Survival analyses showed that PTPRS high expression group has a longer overall survival than PTPRS low expression group (*p* = 0.0184, Figure 1C). Among all dead MPNST patients during follow-up period, the proportion of PTPRS positive cells in IHC stainings was determined using ImageJ. Positive linear regression correlation was detected between length of post-operational survival and proportion of PTPRS positive cells in MPNST (*p* = 0.0003, Figure 1D).

**TABLE 1:** Clinical parameter with PTPRS expression (high = 2–3, low = 0–1).

	PTPRS expression		*p*-Value
	High	Low	
Gender			
Male	17	7	1.000
Female	17	8	
Age			
<45	13	11	0.032*
>45	21	4	
Tumor size			
T1	3	2	0.629
T2	9	5	
T3	3	2	
T4	3	0	
Tumor site			
Head and neck	11	3	0.320
Trunk	9	7	
Limbs	13	4	
NF1			
With	14	4	0.208
Without	15	11	
Survival			
Survive	11	4	0.066
Death	5	9	

*p < 0.05.

### PTPRS Downregulation Prompts MPNST Migration/Invasion via EMT *in vitro* and *in vivo*

**FIGURE 2:**
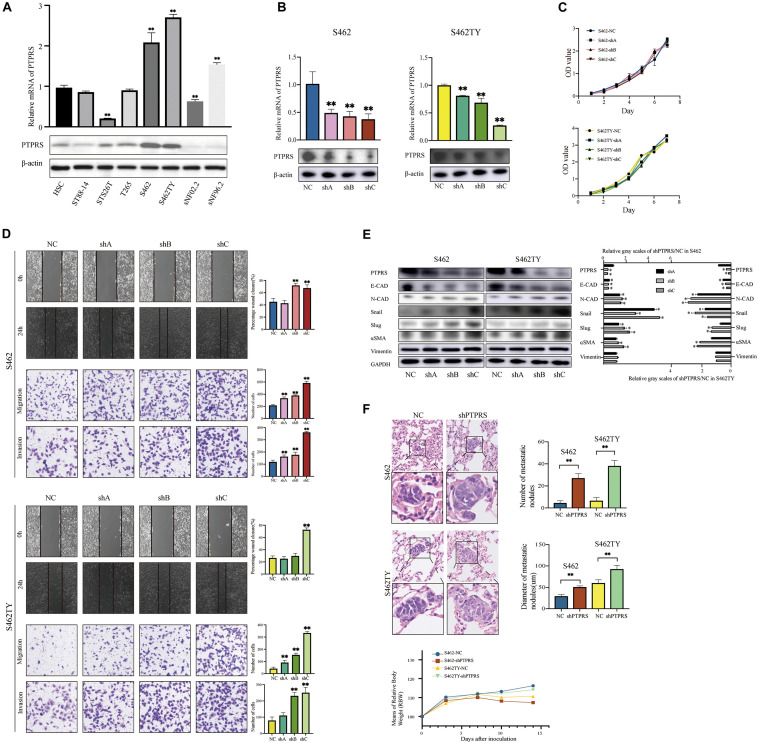
Downregulation of PTPRS mediated MPNST cell migration and invasion *in vitro* and metastasis *in vivo*. **(A)** Relative PTPRS mRNA and protein levels in HSC and MPSNT cell lines. β-Actin was used as a loading control. **p < 0.05. **(B)** Relative PTPRS mRNA and protein levels in PTPRS knockdown cell lines. The shA, shB, and shC represented three hairpins. β-Actin was used as a loading control. **p < 0.05. **(C)** Effects of PTPRS knockdown on cell proliferation (*p* > 0.05). **(D)** Effects of PTPRS knockdown on cell migration and invasion. Representative images of wound healing assays and migrated or invasive cells were shown (left panels). Results were represented as mean ± SD in triplicate using bar graph (right panels). **p < 0.05. **(E)** Protein levels of key EMT markers, including E-cadherin, N-cadherin, Snail, Slug, α-SMA, and vimentin were shown in indicated cells (left panels). Relative gray scales of shPTPRS/NC were shown as grouped columns. Results were represented as mean ± SD in triplicate using bar graph (right panels). *p < 0.05. **(F)** Effects of PTPRS knockdown on tumor metastasis *in vivo*. Left panel: Representative hematoxylin and eosin images of mice lung tissue sections. Magnifications: ×40; ×100. Right panel: Number and diameter of lung metastatic foci in each group (*n* = 8) were presented as mean ± SD. **p < 0.05. Bottom panel: Changes of body weights of mice over time (*p* > 0.05). PTPRS, protein tyrosine phosphatase receptor S; MPNST, malignant peripheral nerve sheath tumor; HSC, human Schwann cell; NC, normal control; E-CAD, E-cadherin; N-CAD, N-cadherin; α-SMA, alpha smooth muscle actin.

Seven MPNST cell lines and one normal Schwann cell line were screened for PTPRS expression by using qPCR and western blottings (Figure 2A). S462 and S462TY were selected as high-PTPRS-expression cell lines. PTPRS down-regulated lentivirus was established as previously described (Wang et al., 2015) and effectiveness was confirmed in S462 and S462TY cell lines (Figure 2B). *In vitro* experiments were carried out by the stable strain constructed by shA, shB, and shC. *In vivo* experiments were carried out by PTPRS shC (which is the most effective lentivirus and referred to shPTPRS in the following manuscript). No difference was detected in proliferation of PTPRS downregulated MPNST cells and control cells (Figure 2C). PTPRS downregulation significantly increased migration and invasion capabilities of S462 and S462TY cells (Figure 2D). Subcutaneous MPNST tumor models revealed similar results *in vivo*. There were no significant differences in tumor growth and mice body weight (Supplementary Figure S1C). However, PTPRS downregulation significantly increased number and size of metastasis nodes (Figure 2F). PTPRS downregulation increased expression of N-cadherin and alpha smooth muscle action (αSMA) as well as decreased expression of E-cadherin (Figure 2E). Immunofluorescence stainings of E-cadherin and vimentin showed similar results (Supplementary Figure S1D). Downstream EMT transcriptional factors, including Snail and Slug, were also activated (Figure 2E). All these findings indicated EMT was enhanced by downregulation of PTPRS.

### PTPRS Overexpression Suppresses MPNST Migration/Invasion via EMT *in vitro*

**FIGURE 3:**
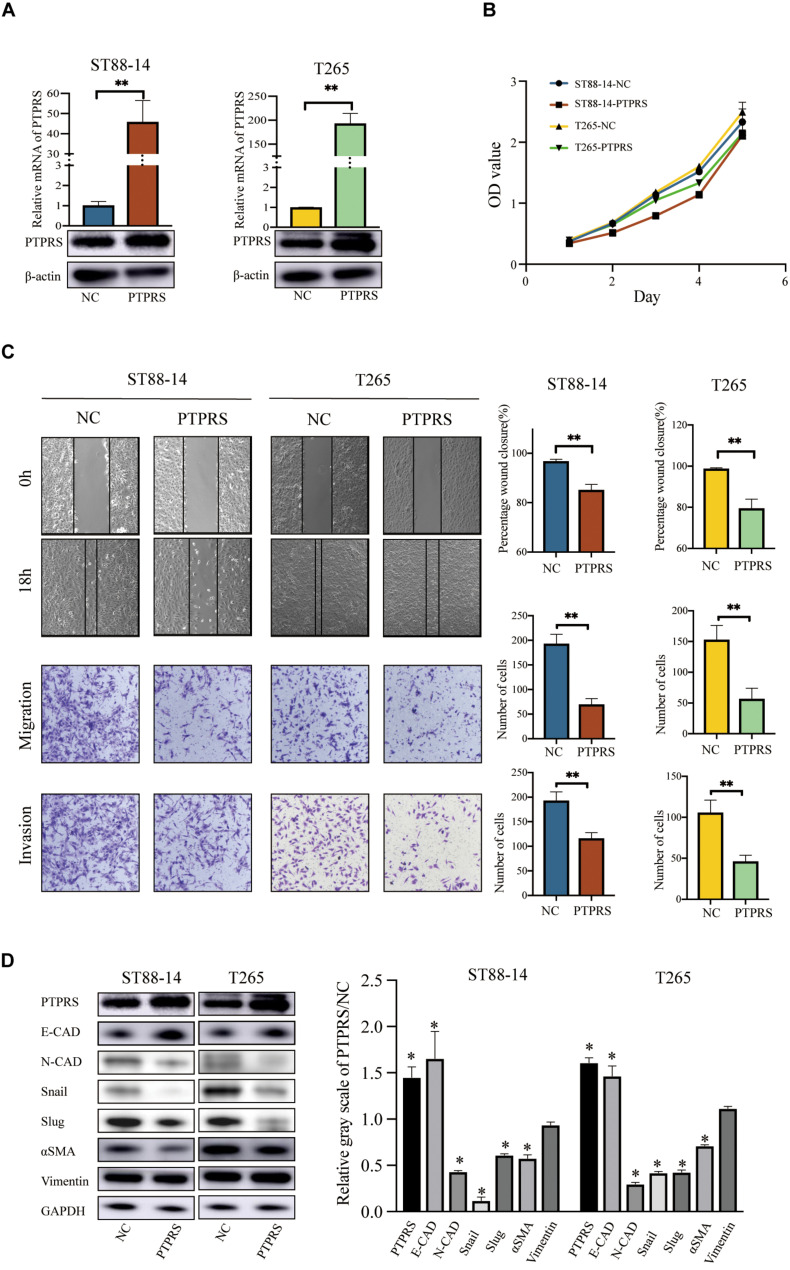
Overexpression of PTPRS mediated MPNST cell migration and invasion *in vitro*. **(A)** Relative PTPRS mRNA and protein levels in PTPRS overexpression cell lines. β-Actin was used as a loading control. **p < 0.05. **(B)** Effects of PTPRS overexpression on cell proliferation (*p* > 0.05). **(C)** Effects of PTPRS overexpression on cell migration and invasion. Representative images of wound healing assays and migrated or invasive cells were shown (left panels). Results were represented as mean ± SD in triplicate using bar graph (right panels). **p < 0.05. **(D)** Protein levels of key EMT markers, including E-cadherin, N-cadherin, Snail, Slug, α-SMA, and vimentin, were shown in indicated cells (left panels). Relative gray scales of PTPRS/NC were shown as grouped columns. Results were represented as mean ± SD in triplicate using bar graph (right panels). *p < 0.05. PTPRS, protein tyrosine phosphatase receptor S; MPNST, malignant peripheral nerve sheath tumor; NC, normal control; E-CAD, E-cadherin; N-CAD, N-cadherin; α-SMA, alpha smooth muscle actin.

MPNST ST88-14 and T265 were selected as PTPRS low expression cell lines for investigations (Figure 3A). As the length of PTPRS coding sequence exceeded in the capacity of lentivirus as previously described (Wang et al., 2015), PTPRS overexpression was realized using plasmid transfections in ST88-14 and T265 cells, which has relatively short effective duration. Therefore, only *in vitro* experiments were performed. PTPRS overexpression had no impacts on MPNST cell proliferation (Figure 3B). Overexpression of PTPRS significantly suppressed the ability of migration and invasion of MPNST cells (Figure 3C). Consistent with previous results, EMT was suppressed in PTPRS overexpressed MPNST cells. E-cadherin was elevated and N-cadherin/αSMA were downregulated (Figure 3D). Expressions of downstream EMT transcriptional factors, Snail and Slug, were significantly decreased.

### Screening of EMT Related ABP in PTPRS Downregulated MPNST Cells

**FIGURE 4:**
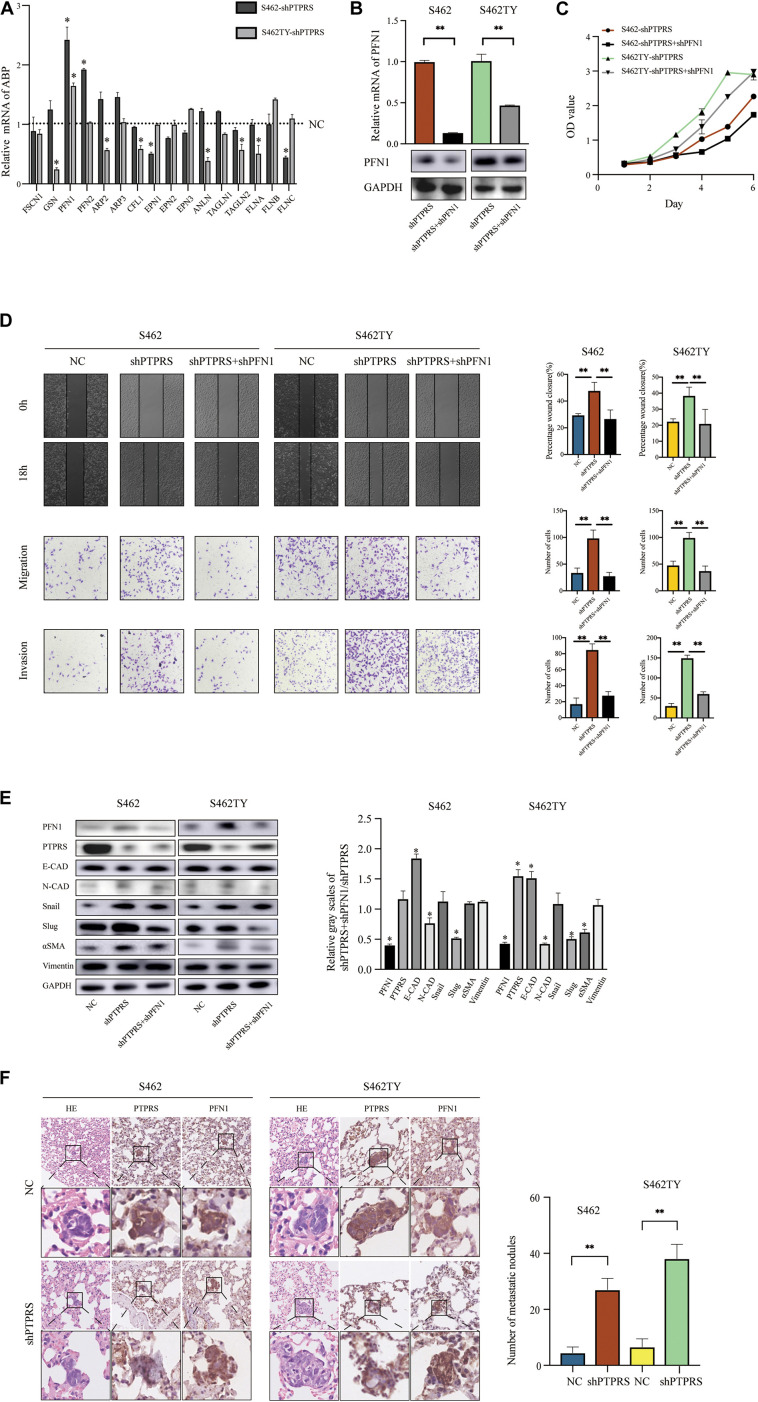
PTPRS mediated MPNST cell migration and invasion via PFN1. **(A)** Relative mRNA levels of EMT related ABP in two PTPRS knockdown cell lines. GAPDH was used as a loading control. *p < 0.05. **(B)** Relative PFN1 mRNA and protein levels in PTPRS knockdown and PTPRS + PFN1 dual knockdown cell lines. GAPDH was used as a loading control. **p < 0.05. **(C)** Effects of PFN1 knockdown on cell proliferation (*p* > 0.05). **(D)** Protein levels of key EMT markers, including E-cadherin, N-cadherin, Snail, Slug, α-SMA and vimentin, were shown in indicated cells (left panels). Relative gray scales of shPTPRS + shPFN1/shPTPRS were shown as grouped columns. Results were represented as mean ± SD in triplicate using bar graph (right panels). *p < 0.05. **(F)** Effects of PTPRS + PFN1 dual knockdown on cell migration and invasion. Representative images of wound healing assays and migrated or invasive cells were shown (left panels). Results were represented as mean ± SD in triplicate using bar graph (right panels). **p < 0.05. **(E)** Representative immunostaining images of PFN1 and PTPRS in mice lung tissue sections. Number and diameter of lung metastatic foci in each group (*n* = 8) were presented as mean ± SD. **p < 0.05. PTPRS, protein tyrosine phosphatase receptor S; EMT, epithelial-mesenchymal transition; NC, normal control; FSCN1, fascin actin-bundling protein 1; GSN, gelsolin; PFN1, profilin 1; PFN2, profilin 2; ARP2, actin related protein 2; ARP3, actin related protein 3; CFL1, cofilin 1; EPN1, epsin 1; EPN2, epsin 2; EPN3, epsin 3; ANLN, anillin actin binding protein; TAGLN1, transgelin 1; TAGLN2, transgelin 2; FLNA, filamin A; FLNB, filamin B; FLNC, filamin C; E-CAD, E-cadherin; N-CAD, N-cadherin; α-SMA, alpha smooth muscle actin.

Epithelial-mesenchymal transition involved organization and dynamics of actin cytoskeleton controlled by a large array of ABP. Through literature review, 16 ABP were previously demonstrated to be involved in mediating EMT processes (Supplementary Table S1). All these ABP were screened using qPCR in PTPRS downregulated S462 and S462TY cells with corresponding control cells. Profilin 1 was demonstrated to be the most significantly elevated ABP in both PTPRS downregulated MPNST cells and the trend is consistent in both cell lines (Figure 4A). While profilin 2, an important member of profilin family, was upregulated in one strain by qPCR, there was no significant difference in its protein expression in both knockdown and overexpression cell lines (Supplementary Figure S1B).

### The Role of Profilin 1 in PTPRS Mediated EMT Processes

To investigate the role of profilin 1 in PTPRS mediated EMT processes, profilin 1 downregulated lentivirus was established and MPNST S462 and S462TY cells with both PTPRS and profilin 1 downregulation were established and validated (Figure 4B, Supplementary Figure S1A). Due to the best effectiveness in PFN1 downregulation, shA (referred to shPFN1 in the following manuscript) was chosen to be applied on S462 and S462TY shPTPRS cell lines. No proliferation differences were detected in PTPRS/profilin 1 downregulated MPNST cells and PTPRS only downregulated MPNST cells (Figure 4C). In addition, by additional downregulation of profilin 1, the increased migration and invasion ability induced by PTPRS downregulation could be overcome (Figure 4D). Characteristic markers and downstream EMT transcriptional factors showed similar results. By downregulation of profilin 1, the EMT processes induced by PTPRS downregulation was restored with increased expression of E-cadherin, decreased expression of N-cadherin and αSMA as well as decreased expression of Snail and Slug (Figure 4E). The negative expression correlation was also found in lung metastasis nodes (Figure 4F).

### High Expression of Profilin 1 Negatively Correlated With Prognosis of MPNST Patients

**FIGURE 5:**
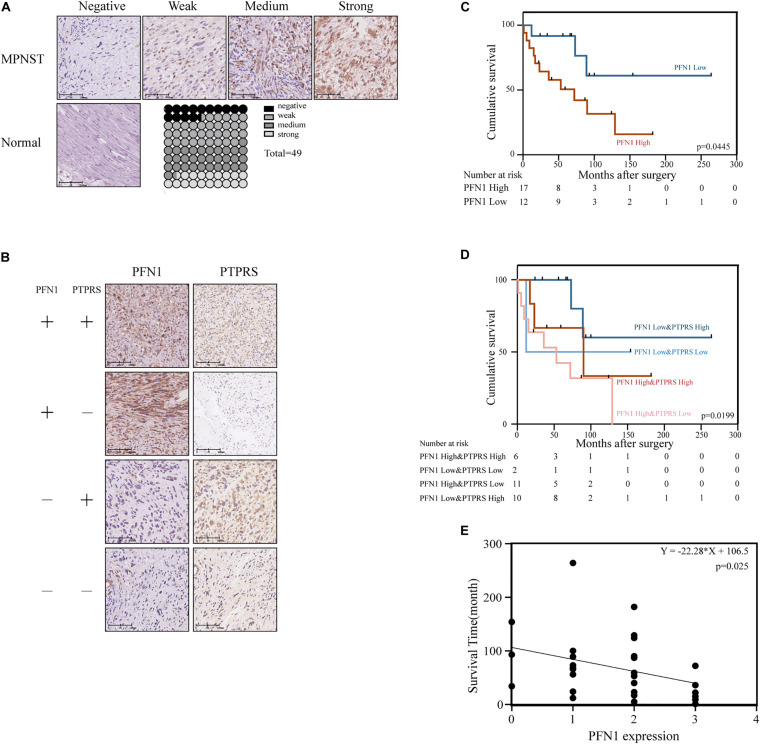
Upregulation of PFN1 correlated with poor prognosis in MPNST patients. **(A)** Representative immunostaining images of PFN1 negative/weak/medium/strong and normal nerve tissue cases and proportions (%) of different levels of tissue microarray dots. **(B)** Representative immunostaining images of PFN1 and PTPRS in same patients. The number of patients fall into each group were 14 (PFN1 high and PTPRS high)/11 (PFN1 high and PTPRS low)/20 (PFN1 low and PTPRS high)/4 (PFN1 low and PTPRS low) in the tissue microarray cohort (total = 49), respectively. The “+” represents high expression (score = 2 or 3); the “–” represents low expression (score = 0 or 1). **(C)** Kaplan–Meier’s curves for cumulative survival according to PFN1 level in MPNST cohort with available follow-up data (*n* = 29, *p* = 0.0445). **(D)** Kaplan–Meier’s curves for cumulative survival according to PFN1&PTPRS level in MPNST cohort with available follow-up data (*n* = 29, *p* = 0.0199). **(E)** Correlation between expression of PFN1 level in tissues of dead patients and survival time after surgery (*p* = 0.0025). PTPRS, protein tyrosine phosphatase receptor S; PFN1, profilin 1; MPNST, malignant peripheral nerve sheath tumor.

The expression of profilin 1 was evaluated in MPNST tissue array and NNT (Figure 5A). Representative figures were shown (Figure 5A). Unlike high PTPRS expression, NNTs showed negative expression of profilin 1. By classifying MPNST patients based on profilin 1 expression (high, 2–3 scores; low, 0–1 scores), clinicopathological correlation analyses revealed profilin 1 expression negatively correlated with large tumor size (*p* = 0.047, Table 2). The representative immunostaining images of profilin 1 and PTPRS in the same patients were shown in Figure 5B. The overall survival of high profilin 1 expression MPNST patients significantly shortened compared with low profilin 1 expression MPNST patients (*p* = 0.0445, Figure 5C and Table 2). When applying both PTPRS and profilin 1 as classification parameters in MPNST patients, the longest overall survival was detected in PTPRS high expression and profilin 1 low expression MPNST patients, while the shortest overall survival was observed in PTPRS low expression and profilin 1 high expression group (*p* = 0.0199, Figure 5D). Negative linear regression correlation was detected between length of post-operational survival and level of profilin 1 expression in MPNST patients (*p* = 0.025, Figure 5E).

**TABLE 2:** Clinical parameter with PFN1 expression (high = 2–3, low = 0–1).

	PFN1 expression		*p*-Value
	High	Low	
Gender			
Male	15	9	0.156
Female	10	15	
Age			
<45	13	11	0.778
>45	12	13	
Tumor size			
T1	3	2	0.047*
T2	7	7	
T3	5	0	
T4	0	3	
Tumor site			
Head and neck	6	8	0.851
Trunk	8	8	
Limbs	9	8	
NF1			
With	7	12	0.372
Without	13	12	
Survival			
Survive	6	9	0.035*
Death	11	3	

*p < 0.05.

## Discussion

Due to local recurrence and early metastasis, the prognosis of MPNST is dismal (Widemann and Italiano, 2018). Even with aggressive surgical resection and combined therapies with chemotherapy and radiotherapy, the improvements for outcomes of MPNST patients remain limited (Kroep et al., 2011; Miao et al., 2019). Therefore, investigation of the molecular mechanisms underlying MPNST recurrence and metastasis is urgently needed. In the present study, we demonstrate that PTPRS is frequently downregulated in MPNST and downregulation of PTPRS significantly increases immigration and invasion by prompting EMT processes via increasing the expression of profilin 1. In addition, downregulation of profilin 1 could substantially restore the impacts of PTPRS on EMT processes. Clinicopathological correlation analyses reveal that low expression of PTPRS and high expression of profilin 1 significantly are associated with disease progression. Survival analyses show PTPRS and profilin 1 could be used as predictors for prognosis with high PTPRS and low profilin 1 indicating the best outcome.

Protein tyrosine phosphatase receptor S was previously demonstrated to play an important role in neural developments and immune responses as well as function as tumor suppressor gene in various types of cancers (Du and Grandis, 2015). Our research team previously identified PTPRS regulated EMT via dephosphorylation of EGFR in hepatocellular carcinoma (Wang et al., 2015). PTPRS–EGFR interactions also played a vital role in chemotherapy resistance in head and neck cancer (Geiger et al., 2016). In addition, STAT3 was another downstream target of PTPRS in cancer progression (Geiger et al., 2016). Although it was well established that ABP had significant impacts on cytoskeleton organization during EMT processes (Kristo et al., 2016; Yin et al., 2019). No potential downstream ABP has been previously screened and identified. Through comprehensively screening all EMT related ABP, profilin 1 was identified.

Profilin 1 is a member of profilin family of small ABP, which ubiquitously expressed in various types of cells (Rodriguez Del Rio et al., 2018). Profilin 1 played an important role in regulation of actin polymerization and cytoskeleton remodeling (Stritt et al., 2018). Serum profilin 1 was independently associated with endothelial dysfunction, cardiovascular events and survival in patients with chronic kidney disease (Eroglu et al., 2017; Li X. et al., 2018). Mutations in profilin 1 led to familiar amyotrophic lateral sclerosis (Daigle et al., 2013). The role of profilin 1 in cancer development and progression has been investigated while heterogeneity still exists. In colorectal cancer and breast cancer, high profilin 1 expression was associated with lower stage and longer survival (Huang et al., 2020). The underlying mechanisms of oncogenic effects of profilin 1 were downstream SMAD3 upregulation and S137 phosphorylation (Tang et al., 2015). However, overexpression of profilin 1 was observed in renal cell carcinoma and gastric cancer (Cheng et al., 2015; Karamchandani et al., 2015). The underlying mechanisms of tumor suppressor effects of profilin 1 were integrin β1/FAK (Cheng et al., 2015). Therefore, the effects and corresponding underlying mechanisms of profilin 1 demonstrated a cancer-dependent manner, which needed to be explored in MPNST.

As local recurrence and metastasis remain the most urgent and challenging obstacles in MPNST treatments and EMT is the key process, our present study elucidates the impacts and underlying mechanisms of PTPRS on EMT from the aspects of profilin 1. These findings add our knowledge about MPNST defining the interaction between PTPRS and profilin 1 in EMT processes. PTPRS and profilin 1 could serve as potential prognostic biomarkers and therapeutic targets for drug development for MPNST patients.

## Data Availability Statement

The raw data supporting the conclusions of this article will be made available by the authors, without undue reservation.

## Ethics Statement

The studies involving human participants were reviewed and approved by Shanghai Ninth People’s Hospital Ethics Committee. Written informed consent to participate in this study was provided by the participants’ legal guardian/next of kin. The animal study was reviewed and approved by Shanghai Medical Experimental Animal Care Commission.

## Author Contributions

Z-CW and Q-FL had full access to all the data in the study and took responsibility for the integrity of the data and the accuracy of the data analysis. Q-FL and Z-CW contributed to the conception of the study. J-YR, Y-HG, X-WC, and M-ML performed the experiments and prepared the manuscript. J-YR and Y-HG wrote the manuscript. X-WC and M-ML contributed significantly to data analysis. WW, C-JW, BG, and H-BZ performed the most of data analyses. J-YR, Y-HG, X-WC, and M-ML helped to perform the analysis with constructive discussions. All authors contributed to the article and approved the submitted version.

## Conflict of Interest

The authors declare that the research was conducted in the absence of any commercial or financial relationships that could be construed as a potential conflict of interest.

## Supplementary Material

Supplementary Figure 1 **(A)** Relative PFN1 mRNA and protein levels in S462 PTPRS knockdown cell line. The shA, shB, shC represent three hairpins. β-actin was used as a loading control. ^∗∗^*p* < 0.05. **(B)** Protein levels of PFN2 in knockdown cell lines and PFN1, PFN2 in overexpression cell lines. **(C)** Xenograft mice models of MPNST cells. Up panels: Photos of xenograft mice models and xenografts. Bottom panel: Changes of tumor volume over time (*p* > 0.05). **(D)** Representative immunofluorescence images of E-cadherin and vimentin in S462-NC and shPTPRS. PTPRS, protein tyrosine phosphatase receptor S; NC, normal control; PFN1, profilin 1; PFN2, profilin 2; MPNST, malignant peripheral nerve sheath tumor.

Supplementary Table 1 List of 16 ABP’ main function and references.

## Abbreviations

a-SMA: alpha smooth muscle actin
ABP: actin-binding protein
ANLN: anillin actin binding protein
ARP2: actin related protein 2
ARP3: actin related protein 3
CFL1: cofilin 1
E-CAD: E-cadherin
EGFR: epithelial growth factor receptor
EMT: epithelial-mesenchymal transition
EPN1: epsin 1
EPN2: epsin 2
EPN3: epsin 3
FLNA: filamin A
FLNB: filamin B
FLNC: filamin C
FSCN1: fascin actin-bundling protein 1
GAPDH: glyceraldehyde 3-phosphate dehydrogenase
GSN: gelsolin
HSC: human Schwann cell
IHC: immunohistochemistry
MPNST: malignant peripheral nerve sheath tumor
N-CAD: N-cadherin
NC: normal control
NF1: neurofibromatosis type 1
NNT: normal nerve tissue
PFN1: profilin 1
PFN2: profilin 2
PTPRS: protein tyrosine phosphatase receptor S
TAGLN1: transgelin 1
TAGLN2: transgelin 2.
