# Supplementary figures and images for: Protein Tyrosine Phosphatase Receptor S Acts as a Metastatic Suppressor in Malignant Peripheral Nerve Sheath Tumor via Profilin 1-Induced Epithelial-Mesenchymal Transition
Source: Front Cell Dev Biol. 2020 Oct 9;8:582220. doi: 10.3389/fcell.2020.582220 (PMC7581944; doi:10.3389/fcell.2020.582220)

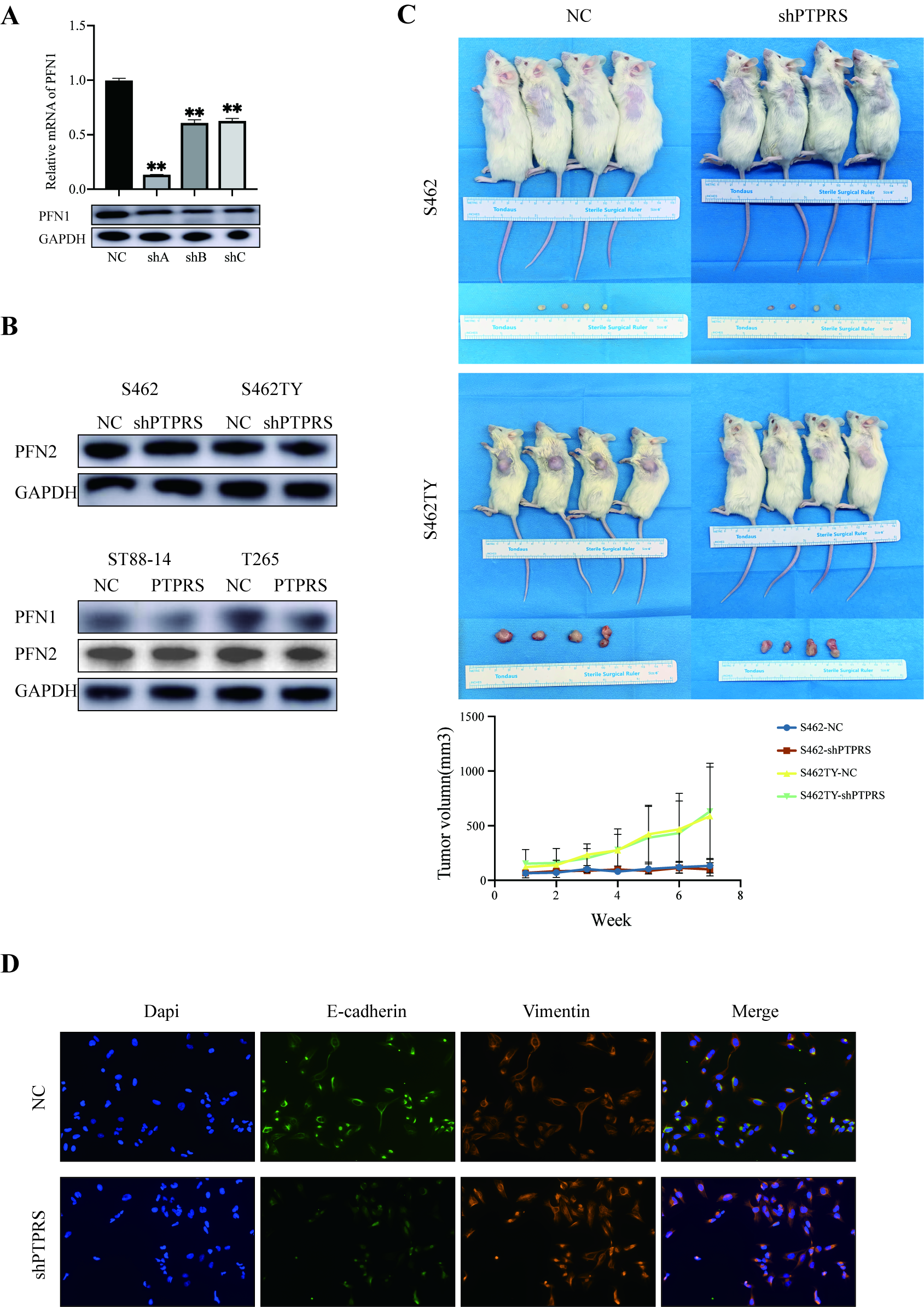

Supplement: Supplementary Figure 1 — (A) Relative PFN1 mRNA and protein levels in S462 PTPRS knockdown cell line. The shA, shB, shC represent three hairpins. β-actin was used as a loading control. ∗∗p < 0.05. (B) Protein levels of PFN2 in knockdown cell lines and PFN1, PFN2 in overexpression cell lines. (C) Xenograft mice models of MPNST cells. Up panels: Photos of xenograft mice models and xenografts. Bottom panel: Changes of tumor volume over time (p > 0.05). (D) Representative immunofluorescence images of E-cadherin and vimentin in S462-NC and shPTPRS. PTPRS, protein tyrosine phosphatase receptor S; NC, normal control; PFN1, profilin 1; PFN2, profilin 2; MPNST, malignant peripheral nerve sheath tumor. [file Image_1.TIF]
