# Supplementary material for: Protein Tyrosine Phosphatase Receptor S Acts as a Metastatic Suppressor in Malignant Peripheral Nerve Sheath Tumor via Profilin 1-Induced Epithelial-Mesenchymal Transition
Source: Front Cell Dev Biol. 2020 Oct 9;8:582220. doi: 10.3389/fcell.2020.582220 (PMC7581944; doi:10.3389/fcell.2020.582220)
Supplement: Supplementary Table 1 — List of 16 ABP’ main function and references. [file Table_1.DOCX]

**SUPPLEMENTARY TABLE 1 List of 16 ABP’ main function and references.**

| Gene | Protein coded | Main function | Main reference 1 | 2 |
| --- | --- | --- | --- | --- |
| FSCN1 | Fascin | a promoter of cancer aggressiveness | Fascin Induces Epithelial-Mesenchymal Transition of Cholangiocarcinoma Cells by Regulating Wnt/β-Catenin Signaling. | Fascin-1 Expression Correlates With Repression of E-cadherin Expression in Hepatocellular Carcinoma Cells and Augments Their Invasiveness in Combination With Matrix Metalloproteinases |
| GSN | Gelsolin | Controversial | Involvement of Gelsolin in TGF-beta 1 Induced Epithelial to Mesenchymal Transition in Breast Cancer Cells | siRNA Gelsolin Knockdown Induces Epithelial-Mesenchymal Transition With a Cadherin Switch in Human Mammary Epithelial Cells |
| PFN1 | Profilin 1 | Controversial; may be concentration-dependent | A Balanced Level of profilin-1 Promotes Stemness and Tumor-Initiating Potential of Breast Cancer Cells | The Role of Profilin Complexes in Cell Motility and Other Cellular Processes |
| PFN2 | Profilin 2 | Controversial | Loss of Profilin 2 Contributes to Enhanced Epithelial-Mesenchymal Transition and Metastasis of Colorectal Cancer | PFN2, a Novel Marker of Unfavorable Prognosis, Is a Potential Therapeutic Target Involved in Esophageal Squamous Cell Carcinoma |
| ARP2 | actin related protein 2 | They act as a ARP2/3 complex, may be a promotor | Formin-dependent TGF-β Signaling for Epithelial to Mesenchymal Transition | Exo70 Isoform Switching Upon Epithelial-Mesenchymal Transition Mediates Cancer Cell Invasion |
| ARP3 | actin related protein 3 |  |  |  |
| CFL1 | Cofilin 1 | a promoter of cancer aggressiveness | Cofilin-1 Signaling Mediates Epithelial-Mesenchymal Transition by Promoting Actin Cytoskeleton Reorganization and Cell-Cell Adhesion Regulation in Colorectal Cancer Cells | Comparison of cofilin‑1 and Twist‑1 Protein Expression in Human non‑small Cell Lung Cancer Tissues |
| EPN1 | Epsin 1 | a promoter of cancer aggressiveness | The Epsin Family of Endocytic Adaptors Promotes Fibrosarcoma Migration and Invasion | Motif Mimetic of Epsin Perturbs Tumor Growth and Metastasis |
| EPN2 | Epsin 2 |  |  |  |
| EPN3 | Epsin 3 |  | A Self-Sustaining Endocytic-Based Loop Promotes Breast Cancer Plasticity Leading to Aggressiveness and Pro-Metastatic Behavior | Overexpression of Epsin 3 Enhances Migration and Invasion of Glioma Cells by Inducing epithelial‑mesenchymal Transition |
| ANLN | Anillin | a promoter of cancer aggressiveness | Knockdown of Anillin Actin Binding Protein Blocks Cytokinesis in Hepatocytes and Reduces Liver Tumor Development in Mice Without Affecting Regeneration | Anillin Regulates Breast Cancer Cell Migration, Growth, and Metastasis by Non-Canonical Mechanisms Involving Control of Cell Stemness and Differentiation |
| TAGLN1 | Transgelin 1 | Controversial | TAGLN Expression Is Upregulated in NF1-associated Malignant Peripheral Nerve Sheath Tumors by Hypomethylation in Its Promoter and Subpromoter Regions | Expression of Cofilin-1 and Transgelin in Esophageal Squamous Cell Carcinoma |
| TAGLN2 | Transgelin 2 | a promoter of cancer aggressiveness | Hypoxia-inducible Transgelin 2 Selects Epithelial-To-Mesenchymal Transition and γ-radiation-resistant Subtypes by Focal Adhesion Kinase-Associated Insulin-Like Growth Factor 1 Receptor Activation in Non-Small-Cell Lung Cancer Cells | TAGLN2 Is a Candidate Prognostic Biomarker Promoting Tumorigenesis in Human Gliomas |
| FLNA | Filamin A | a promoter of cancer aggressiveness | Filamin A Upregulation Correlates With Snail-induced Epithelial to Mesenchymal Transition (EMT) and Cell Adhesion but Its Inhibition Increases the Migration of Colon Adenocarcinoma HT29 Cells | Zinc-α2-glycoprotein 1 Promotes EMT in Colorectal Cancer by Filamin A Mediated Focal Adhesion Pathway |
| FLNB | Filamin B | Controversial | An Alternative Splicing Switch in FLNB Promotes the Mesenchymal Cell State in Human Breast Cancer | Targeting Filamin B Induces Tumor Growth and Metastasis via Enhanced Activity of Matrix metalloproteinase-9 and Secretion of VEGF-A |
| FLNC | Filamin C | Controversial | Testin and filamin-C Downregulation by Acetylated Siah2 Increases Invasiveness of Helicobacter Pylori-Infected Gastric Cancer Cells | High filamin-C Expression Predicts Enhanced Invasiveness and Poor Outcome in Glioblastoma Multiforme |
